# International Otological Congress

**Published:** 1888-10

**Authors:** 


### INTERNATIONAL OTOLOGICAL CONGRESS.

The Fourth International Otological Congress was held in Brussels, Belgium, September.

There was a large attendance of representative otologists, and the many countries represented made it markedly international in character. Several American otologists were present. Dr. Ch. Delstanche, of Brussels, presided.

Professor GRADANGA, of Padua, Italy, made a report of extensive investigations on the *development of the auricle.*

Professor POLITZER, of Vienna, Austria, reported the results of investigation of the pathology of middle-ear affections.

Dr. RÖHRER, of Zurich, Switzerland, in a paper presented, discussed "Rume's Experiment in Diseases of the Labyrinth."

Dr. GELLÉ, of Paris, France, gave a description of his researches on binaural reflexes.

President DELSTANCHE read a paper on "The Treatment of Sclerosis of the Middle Ear."

Dr. BARR, of Glasgow, Scotland, described some of Dr. Macewen's operations of trephining in cases of cerebral abcesses, and drew attention to possible advantages in such cases arising in ear diseases involving the brain.

Dr. BABER, of Brighton, England, presented the subject of nose and ear diseases produced or aggravated by excessive use of alcoholic beverages.

The fifth congress is to be held in Florence, Italy, in 1892.

Questions proposed for discussion in the next congress include—pathological anatomy of the internal ear, diagnosis and treatment of abscesses of the brain due to diseases of the ear, and constitutional treatment in ear disease.

